# Internally Self-Assembled Pickering Mesosomes Stabilized by Positively Charged Lactoferrin

**DOI:** 10.1021/acs.langmuir.5c02484

**Published:** 2025-11-03

**Authors:** Yi Li, Brent S. Murray, Célia Ferreira, Francisco M. Goycoolea, Amin Sadeghpour

**Affiliations:** † Food Colloids and Bioprocessing Research Group, School of Food Science and Nutrition, 4468 University of Leeds, Leeds, West Yorkshire LS2 9JT, United Kingdom; ‡ Department of Cell Biology and Histology, Faculty of Biology, University of Murcia, Campus de Espinardo, Murcia 30100, Spain

## Abstract

Mesosomes as sub-micrometer-sized emulsions containing internally self-assembled lyotropic liquid crystalline (LLC) mesophases were engineered using lactoferrin (LF) as a Pickering stabilizer. LF was pretreated with ultrasonication to prevent agglomeration and applied under controlled pH conditions during processing, resulting in emulsions with a positive surface charge and high stability. Glycerol monooleate (GMO) in mixtures with oleic acid (OA) at varying concentration ratios were used to produce four different lyotropic mesophases: bicontinuous cubic (*Pn*3*m*), inverse hexagonal (H_2_), inverse micellar cubic (*Fd*3*m*), and microemulsions (ME). At a preadjusted pH of 4, LF stabilized all four lyotropic mesophases via a Pickering-type mechanism, forming mesosomes with hydrodynamic diameters (*d*_H_) ranging from ∼187 to ∼239 nm, as measured by dynamic light scattering (DLS). High-pressure ultrasonication was used to drive positively charged LF to adsorb at the LLC–water interface, confirmed via confocal microscopy. The sonication led to a distinct positive surface charge on the mesosomes, as evidenced by the ζ potential of ∼+30 mV, without compromising the mesosomes’ size and stability. Time-resolved DLS experiments showed that all mesosomes had long-term stability against coalescence and aggregation for at least 1 month of storage at room temperature. Small-angle X-ray scattering was used to study the phase behavior of GMO/OA mixtures before and after their stabilization by LF. The results showed that all four bulk mesophases maintained their structural hierarchy after being homogenized into mesosomes. Notably, pure GMO stabilized by LF formed cubosomes of diamond *Pn*3*m*, the same morphology as GMO in the bulk. This behavior is different from what has been observed for the commonly used stabilizer for LLC systems, Pluronic F127, which induces a transition to a primitive *Im*3*m* cubic morphology upon stabilization.

## Graphical abstract

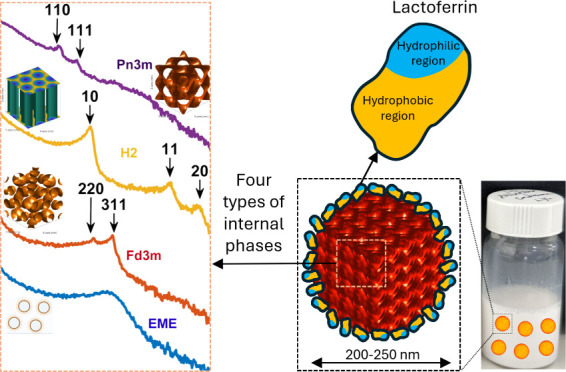

## Introduction

The engineering of nanostructured emulsions with internally self-assembled architectures (mesosomes) has received significant attention in colloid and interface science over the past decades. Unlike conventional oil-in-water emulsions, mesosomes exhibit unique internal hierarchies from lyotropic liquid crystals (LLCs) that impart superior encapsulation and controlled release properties.^1–5^ LLCs form via self-assembly of amphiphilic lipid molecules of specific shapes in an excess solvent (typically water)^6^ due to the molecules’ hydrophobic–hydrophilic balance.^7^ Depending on the lipid types (and molecular shapes), compositions, and physical conditions, various liquid crystalline mesophases can be formed with varying surface curvatures.^8,9^ The most common LLC mesophases are made of different combinations of monoglycerides, such as glycerol monooleate (GMO) plus oils, including fatty acids or triglycerides.^10^ They include the inverse hexagonal (H_2_), micellar cubic phase (*Fd*3*m*), microemulsions (ME), and bicontinuous cubic phases. Usually, three different lattice types make up the reverse bicontinuous cubic phases: the primitive lattice (*Im*3*m*), the double-diamond lattice (*Pn*3*m*), and the gyroid lattice (*Ia*3*d*). The advanced structural and functional properties position LLCs as promising systems for applications across food innovation, biotechnology, and nanomedicine. However, stabilizing these gel-like self-assemblies is more challenging than stabilizing the simple oil phases of classical emulsions.^11^ Conventional surface-active agents are often unsuitable due to the structural rigidity and complex hydrophilic–lipophilic balance of the mesophases. The common stabilizers of LLCs are triblock copolymers, e.g., Pluronic F127 and its homologues, which can form sub-micrometer-sized mesosomes.^12,13^ However, Pluronics often interfere with self-assembling molecules, resulting in a change in LLC hierarchy,^14,15^ for example, altering the bulk *Pn*3*m* phase to *Im*3*m* in dispersion.^9,16,17^ LLCs stabilized by solid nanoparticles (Pickering particles) are less common but often do not change the internal structure, probably due to the significantly larger size of the particles and their different chemical character compared to the self-assembling lipids. For instance, 0.5 wt % Laponite clay particles and nanosheets stabilized 5 wt % monoolein-based LLCs, resulting in the formation of mesosomes of cubic and hexagonal phases.^9,18,19^ Similarly, hydrophilic silica particles modified with oleic acid (OA) have demonstrated strong stabilizing capabilities against all four LLC phases, resulting in sub-micrometer-sized droplets.^20^

In Pickering emulsions, solid particles strongly adsorb at the oil–water interface, providing the emulsions with high stability against coalescence.^21–30^ The detachment energy of solid particles at an interface can be calculated by the following equation:^20,31^

1 $$\Delta E=-\pi R^{2}\gamma_{\mathrm{ow}}{\left(1-\left|\cos\theta \right|\right)}^{2}$$

where *R* is the particle radius, *θ* is the contact angle of particles at the interface, and *γ*_ow_ is the interfacial tension between the two phases. The particle radius exhibits a quadratic influence on Δ*E*, making it a critical parameter in determining the stability of Pickering emulsions. The contact angle also influences the stability of emulsions, with the highest detachment energy occurring at a contact angle of 90°. Therefore, the energy required to detach a solid particle from an interface can be thousands of times greater than the thermal energy (*k*_B_ *T*).^32^ In other words, in Pickering emulsions, particles are irreversibly anchored at the interface, creating a long-lasting barrier against coalescence and Ostwald ripening.^33–35^ It is empirically known that the diameter of the Pickering particles should be at least 10 times smaller than that of the emulsion droplets.^36^ Hence, obtaining sub-micrometer-sized emulsions requires Pickering particles with an average diameter in the range of a few tens of nanometers. The stability and biocompatibility of Pickering emulsions can be further enhanced if globular proteins can be used as Pickering stabilizers. The extent to which proteins retain their globular structure at an interface determines their Pickering stabilization capacity and the long-term stability of the resulting emulsions. We note that, should nonspherical proteins or aggregates adsorb at the interface, the quantitative description of detachment energy would differ from eq 1.^31,37^ Previous studies have demonstrated the simple oil-in-water stabilizing effect of a range of relatively insoluble proteins and their aggregates, e.g., pea proteins.^38–42^ Sarkar and co-workers reported oil-in-water (O/W) Pickering emulsions stabilized by pea protein isolates (PPI) with droplet sizes of ∼25 μm and several months of stability.^43^ Pea protein complexes with phenolic compounds have also been demonstrated to form surface active nanoparticles, resulting in improved emulsion stability by about 20% compared to PPI and a reduced droplet size (∼10 μm).^44,45^ In another study, a reduced droplet size (2.3 μm) was achieved by pea protein isolates in the form of nanoparticles (134–165 nm) at pH 3.0.^46^ Pickering emulsions offer advantages beyond physical stability, notably a lower cytotoxicity^47–51^ and enhanced resistance to enzymatic digestion. A strong interfacial layer formed by solid particles limits the access of enzymes to lipids within droplets, thereby reducing the rate of lipid digestion.^52^ Zhang et al. demonstrated the digestive resistance of Pickering O/W emulsions in a system stabilized by soy protein isolate–bacterial cellulose nanofibril complexes.^53^

Despite providing high stability against coalescence, many Pickering formulations fail to provide stability against aggregation, due to the excessive hydrophobic nature of their interfacial layer. While a certain level of hydrophobicity is necessary to promote particle adsorption at the interface, introducing surface charges or steric hindrance can help prevent aggregation. Hence, recent advances have centered on formulating Pickering emulsions exhibiting electrostatic stability via adsorption of charged particles at the interface.^54–56^ Such electrostatic stability not only prevents aggregation but also enables the release of encapsulating materials in emulsions under various physicochemical conditions.^57,58^ Binary mixtures of oppositely charged particles or molecules have been shown to modulate the overall charge and hydrophobicity at the oil–water interface, thereby enabling control over interfacial coverage and droplet size.^20,59,60^

Lactoferrin (LF), primarily extracted from milk but also obtainable from sustainable sources such as microbial systems,^61^ is a glycoprotein with a molecular weight (*M*_w_) of 80 kDa. It has a tertiary structure that comprises 16 intramolecular disulfide bonds that greatly stabilize its conformation.^62–65^ The cationic amino acid residues in the N-terminal region (N1 domain, residues 1–50 ^66^) make LF highly positively charged at neutral pH. The specific tertiary structure of LF also features hydrophobic domains that introduce sufficient amphiphilic character to the molecules,^67^ making them interfacially active and ideal for stabilizing oil–water interfaces. Studies have reported the stabilization of 20–30% oil into cationic droplets of approximately 0.5 μm via LF adsorption at the oil–water interface, with stability maintained across a pH range of 3.0–7.0.^67,68^ McClements and colleagues conducted a comprehensive stability study under various physicochemical conditions, demonstrating that LF imparts high stability to emulsions at temperatures below 60 °C, corresponding to the protein’s denaturation point. The stability of emulsions against salt was also maintained at ionic strengths below 200 mM.^69^ Another interesting study demonstrated that LF’s binding affinity to mucin enhances its emulsion stability in artificial saliva,^70^ whereas the emulsions become susceptible to aggregation under intestinal bile and pH conditions.^71^

Several studies have also explored the formulation of LF conjugates with other biopolymers and proteins to develop multifunctional emulsions, such as those with added antioxidant properties. These conjugates often lead to the formation of micrometer-sized Pickering emulsions.^72^ For instance, complexes formed by electrostatic interactions between positively charged LF and negatively charged fucoidan offer excellent emulsion stability, making them effective for bioactive encapsulation and extending shelf life.^73^ Similarly, an improved lipid oxidation resistance was achieved in LF-cellulose nanocrystal (CNC) stabilized emulsions at both pH 3.0 and 7.0.^74^ In addition to polysaccharide interactions, LF has been incorporated into dual-protein nanoparticle systems. Wang et al. reported that LF–zein complex nanoparticles at a 1:1 ratio effectively balanced electrostatic and hydrophobic interactions, resulting in the formation of a cohesive and viscoelastic film at the emulsion interface.^75^ Another study showed that Pickering emulsions stabilized by lactoferrin and inulin complexes exhibited delayed digestion of lactoferrin at the O/W interface.^65^

Despite its broad functional versatility, LF has not been investigated as a stabilizer of mesosomes with internal self-assembled LLCs. Moreover, mesosomes in general lack a diverse range of effective stabilizers, particularly those capable of tailoring their surface properties (e.g., surface charge). Boyd and colleagues have reported one of the very few works that utilize proteins (in their case β-casein) to effectively stabilize GMO-based cubosomes of the *Pn*3*m* phase.^76^ They concluded that the protein provides steric stabilization, preventing internal phase transitions within the cubosomes. In the current study, we present the first demonstration of LF stabilizing sub-micrometer-sized droplets of internally hierarchical LLCs, characterized by high electrostatic stability via the LF-imparted positively charged surface. Our approach enables the design of mesosomes with bioadhesive properties, facilitating the control of electrostatic interactions with negatively charged biointerfaces. Specifically, our goal is to deploy these mesosomes as targeted delivery vehicles to mucin-rich domains within the gastrointestinal tract. Beyond stability and surface properties, LF’s exceptional iron-binding capacity (with an affinity twice that of transferrin) enables effective iron sequestration, thereby inhibiting pathogen proliferation.^77^ As a pivotal component of the innate immune system, LF exhibits diverse physiological functions, including immunomodulation, anti-inflammatory activity, and regulation of cellular growth. The protein’s biological versatility is facilitated by its ability to interact with multiple cellular receptors and signaling pathways.^78^ The incorporation of all the above biochemical and physiological properties of LF into a new emulsion system enables innovative applications in food, bioengineering, and nanomedicine.

## Materials and Methods

### Materials

Lactoferrin (LF) with ≥95% purity (*M*_w_, 80000 Da; density, 1.48 ± 0.10 g/cm^3^; iron-rich hololactoferrin) was purchased from BOC Sciences (London, U.K.). Glycerol monooleate (GMO) (purity > 92%) was generously provided by Croda (Snaith, U.K.). Oleic acid (OA) with a purity of 90% was purchased from Sigma-Aldrich (Poole, U.K.). All chemicals were utilized as received, without further purification. Unless otherwise stated, solutions and dispersions were prepared with ultrapure water (a resistivity of 18.2 MΩ cm at 25 °C) purified by Merck Millipore (Darmstadt, Germany). The pH adjustment was carried out using solutions (1 or 0.1 M, chosen based on experimental suitability) of hydrochloric acid (37%) and sodium hydroxide (98%), purchased from Sigma (Stenheinm, Germany). For LF characterization, independent samples were freshly prepared at each target pH value (3.0–11.0).

### Lactoferrin Solution Preparation

A 1 wt % LF solution was prepared by dissolving 0.1 g of LF powder in 10 mL of water and then vortex mixing at 2500 rpm for 15 s. The 1 wt % LF solution was then subjected to ultrasonication using a VCX 750 Vibra-Cell (Sonics & Materials, Newtown, CT) at room temperature, with a sonication power of 30% for 30 s, using a probe sonicator in continuous mode. This step was applied to reduce LF aggregation and achieve a uniform particle distribution before using the LF for emulsion preparation.

### Fabrication of Pickering Mesosomes

Mixtures of GMO and OA were used to prepare the lyotropic phase prior to their stabilization. The ratio of GMO/OA, which was varied to form different LLCs, represented by the parameter δ:^20^

2 $$\delta =\frac{\mathrm{GMO}/\left(\mathrm{wt}\%\right)}{\mathrm{GMO}/\left(\mathrm{wt}\%\right)+\mathrm{OA}/\left(\mathrm{wt}\%\right)}\times 100$$

Four distinct δ values of 100, 90, 60, and 54 were selected based on well-established phase diagrams for these mixtures.^8,79,80^ Pure GMO (δ = 100) is known to form cubic phases (*Pn*3*m*) at 25 °C in excess water.^81^ As the OA content increases (decreasing δ), the system transitions through an inverse hexagonal phase (H_2_) at δ = 90, to a micellar cubic phase (*Fd*3*m*) at δ = 60, and a microemulsion (ME) phase at δ = 54.

To prepare 10 wt % mesosomes with diverse internal LLC phases, a systematic sequential addition protocol was applied. First, 9 g of 1 wt % LF solution was added, followed by 1 g of the desired GMO/OA mixture in a molten state (preheated at 55 °C). The resulting 10 wt % lipid–water mixture was then homogenized by tip ultrasonication for 6 min at 40% amplitude in a pulsed mode (3 s ON and 1 s OFF), resulting in a milky dispersion with a faintly perceptible bluish hue. It was noted that the H_2_ and *Pn*3*m* phases were only partially stabilized, with portions of the lyotropic phase remaining as nonstabilized. Quantitative evaluation indicated that 9.65 and 9.45 wt % of the total GMO/OA mixture were stabilized in H_2_ and *Pn*3*m* cases, respectively, while all 10 wt % mixture was emulsified in ME and *Fd*3*m* cases. Water was filtered through a 0.45 μm filter (33 mm diameter) before emulsion preparation, and the pH of the water was monitored using an Orion Star A215 pH/conductivity benchtop multiparameter meter.

### Dynamic Light Scattering (DLS)

The droplet size distributions of all emulsions were characterized using a Malvern Zetasizer Nano ZS (Malvern Instruments Ltd., U.K.) equipped with Zetasizer software (Version 7.11). To ensure optimal measurement conditions and minimize multiple scattering effects, the emulsions were diluted 1000-fold using water preadjusted to pH 4.0, followed by filtration through a 0.45 μm membrane filter. Disposable PMMA cuvettes (capacity, 1.5–3 mL) were procured from Fisher Scientific (Loughborough, U.K.). DLS measurements were conducted at a laser wavelength of 633 nm, scattering angle of 173°, and a temperature of 25 °C. The refractive index (RI) of the internal lipid phase was set to 1.436. Z-average diameter was deduced from the intensity-weighted size distribution, whereas all measurements were repeated three times to ensure the reproducibility of the results.

### Electrophoretic Measurements

ζ potential measurements were performed using a Zetasizer Nano ZS instrument (Malvern Panalytical Ltd., Worcestershire, U.K.). For each sample, a minimum of three independent measurements were performed, with each measurement comprising 100 subruns. All analyses were conducted at a controlled temperature of 25 °C, utilizing the disposable folded capillary cells (DTS1070) from Malvern Panalytical (Worcestershire, U.K.). The emulsions were diluted 100-fold using pH 4.0 water (filtered through a 0.45 μm membrane) before measurements.

### Circular Dichroism (CD)

CD spectroscopy was employed to investigate changes in the secondary structure of LF particles following ultrasonication. Sample preparation followed the same procedure as outlined in the LF preparation. CD measurements were performed in the wavelength range 190–260 nm at a temperature of 25 °C in a 1 mm path length cuvette using a Chirascan Plus spectrophotometer (Applied Photo Physics, Surrey, U.K.). Quantitative fitting of the data was performed using OriginPro 2024 (OriginLab Corp., USA) software, employing its built-in curve fitting functions.

### Small-Angle X-ray Scattering (SAXS)

SAXS experiments were performed to elucidate the internal structures of the self-assembled mesosomes. A SAXSpace camera (Anton Paar, Graz, Austria) equipped with a laboratory-based Cu Kα radiation, providing a wavelength (λ) of 1.5406 Å, and the instrument was equipped with a 1D Mythen detector (Dectris AG, Baden, Switzerland). The sample to detector distance (SDD) was calibrated using a silver behenate standard sample prior to the measurements. All measurements were carried out at 317 cm SDD, providing an accessible *q*-range between 0.05 and 5 nm^–1^, where $q=\frac{4\pi }{\lambda }\sin\left(2\theta \right)$ is the magnitude of the scattering vector, and 2θ is the scattering angle. The temperature was controlled at 20 °C. A semitransparent beam stop was utilized to measure the direct beam intensity (*I*_0_), facilitating subsequent data reduction and background subtraction.^82,83^ Samples were loaded into quartz capillary tubes with an outer diameter of 1.5 mm (Capillary Tube Supplies Ltd., Cornwall, U.K.) and then vacuum sealed.

Phase identification was confirmed using the linear plots of at least three observed diffraction peaks against the theoretical peak positions from liquid crystalline phases.^84,85^ The lattice parameters were determined from the corresponding slopes of the linear relationship between the relative q values and the observed q values in the SAXS pattern (see Supporting Information Figure S1). For instance, the lattice parameter for the H_2_ phase, which represents the distance between adjacent cylinders, was calculated by establishing a linear function where the *x*-axis corresponds to the relative q values obtained theoretically from the Miller indices of the diffraction peaks, and the *y*-axis represents the observed *q* values in SAXS experimental pattern.^85^ The slope of this linear function was then used to calculate the lattice parameter for each phase, as outlined in Table 1 (note, the linear observation in the aforementioned plot itself is an indication of correct phase identification). A different approach was applied for pure LF characterization by SAXS, where the pair distance distribution function (PDDF) was computed using PCG software (University of Graz, Austria). The PDDF represents a histogram of pair distances within a protein particle, weighted by the electron density contrast relative to the solvent and produced by indirect Fourier transformation (IFT).^86,87^ The PDDF was used to determine the shape of proteins in solution and to calculate their radius of gyration (*R*_g_). Additionally, the SAXS data obtained from LF were also fitted using the classical Guinier analysis^88^ to extract *R*_g_ values, which were subsequently compared to those derived from IFT method.

**Table 1:** Summary of Diffraction Peaks Obtained from Four LLC Phases of Lactoferrosomes and Their Calculated Lattice Parameters and Emulsion Characteristics

Pickering mesosomes	Miller indices	Obsd *q* value in SAXS (nm^–1^)	Lattice param from SAXS (nm)	Amount of oil stabilized (wt %)	δ value	*d*_H_ from DLS (nm)
Bicontinuous cubic phase (*Pn*3*m*)	[110]	0.84	10.5 ± 0.009	9.45	100	187 ± 2
	[111]	1.03				
Hexosomes (H_2_)	[10]	1.38	4.5 ± 0.009	9.65	90	201 ± 3
	[11]	2.41				
	[20]	2.78				
Micelle cubic phase (*Fd*3*m*)	[220]	1.19	14.9 ± 0.002	10	60	239 ± 3
	[311]	1.39				
Microemulsion (ME)	Peak position (nm^–1^), 1.5		*d*-spacing (nm), 4.19	10	54	221 ± 2

### Confocal Laser Scanning Microscopy (CLSM)

A Zeiss LSM 800 inverted confocal microscope (Carl Zeiss MicroImaging GmbH, Jena, Germany) was utilized to examine the localization of LF in the emulsions . Fast Green and Nile Red fluorescent stains were used to label the LF and lipid components of the emulsions, respectively. In the final emulsion formulation, their concentrations were adjusted to 1 × 10^–4^ and 4 × 10^–4^ wt %, respectively. The emulsion samples (10 wt % mesosome phase) were prepared following a procedure similar to that used for Pickering mesosome preparation, with the only modification being a reduced ultrasonication time of 30 s. This reduced homogenization time results in the formation of large droplets (10–30 μm in diameter), making the droplet more visible for confocal microscopy analysis. The dyes were mixed by vortexing with the emulsion samples for 3 – 5 min, then left for 15 min before pipetting onto a welled microscope slide and then sealed with a coverslip. Fast Green was excited at 633 nm and Nile Red at 488 nm; images were obtained using a 63× (numerical aperture = 1.4) oil immersion objective lens at room temperature. Image analysis was conducted using ImageJ software.

### Statistical Analysis

All quantitative analyses were performed in triplicate, and data are presented as mean and standard deviation (SD). Statistical analyses were conducted using OriginPro 2024 (OriginLab Corp., USA).

## Results and Discussion

### Characterization of LF

LF was characterized before using it for the stabilization of emulsions. The results in Figure 1 demonstrate the average hydrodynamic diameter (*d*_H_) of LF, which remains constant across pH < 8.5. At pH 4, LF appears to be highly stable with an average hydrodynamic diameter of 43 nm prior to any pretreatment. A significant increase in particle diameter was observed at pH 8.5, with the *d*_H_ reaching a maximum of 800 nm. This dramatic increase in size coincides with the isoelectric point (IEP) of LF,^62,89,90^ where the reduction in net charge results in a decrease in electrostatic repulsion and an increase in net attraction forces between protein molecules, accompanied by their aggregation at around IEP.^65^ This was corroborated by corresponding measurements of ζ potential, also shown in Figure 1. In acidic pH, the ζ potential is positive, measuring approximately 30 mV at pH = 4. As the pH increases, the mobility gradually decreases, reaching zero at pH 8.5. Beyond this point, it continues to decline, becoming negative at values greater than 8.5.

**Figure 1:**
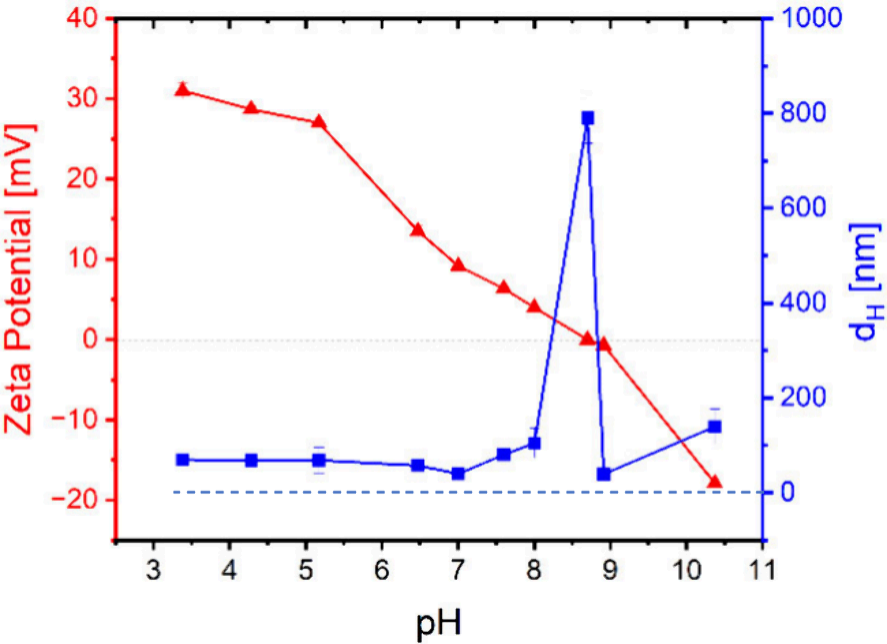
Relationship between pH and the ζ potential and hydrodynamic diameter of LF. The left *y*-axis (red) represents ζ potential(▲), and the right *y*-axis indicates hydrodynamic diameter *d*_H_ (■).

A closer examination of size distribution measurements from DLS reveals three distinct peaks for LF in acidic conditions without prior treatment. To obtain a more uniform size distribution for LF in solution, ultrasonication was applied. After sonication, a bimodal size distribution is observed with peaks appearing at 7.5 and 52 nm. The peak around 52 nm clearly indicates the existence of LF aggregates in the solution, whereas the peak at 7.5 ± 0.2 nm is assigned to monomeric LF.^89–91^ Figure 2 shows the particle size distributions before and after sonication (as described in Materials and Methods) at pH = 4. We note that aggregation in protein systems is common, due to hydrophobic or van der Waals interactions or electrostatic attractions between oppositely charged regions of protein molecules.^25,92^ It is of great importance to ensure that the protein particles used to stabilize the emulsions are much smaller than the intended oil droplet size, and sonication appears to be an effective approach to reduce the size and polydispersity of LF.

**Figure 2:**
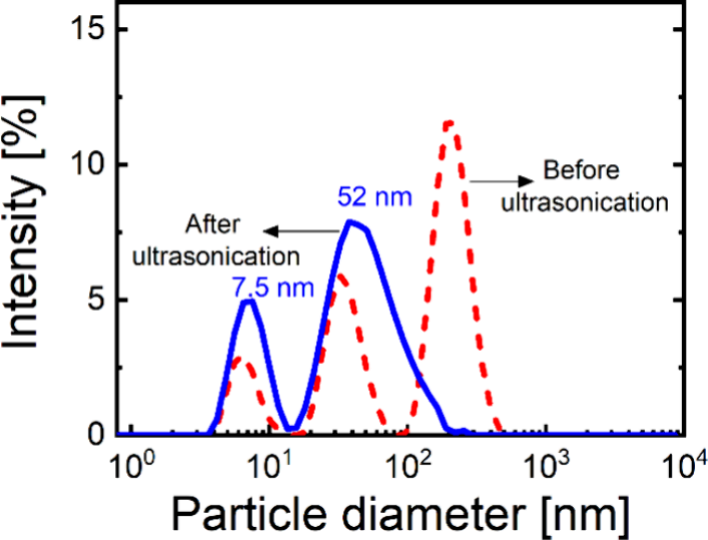
Intensity-weighted size distribution of LF before (dashed line ) and after (solid line) ultrasonication at pH 4. The three-peak distribution observed without ultrasonication transforms into a bimodal distribution following ultrasonic treatment, with peaks appearing at 7.5 and 52 nm.

To gain more detailed insights into the molecular shape and secondary structures of LF, further ultrastructural characterization was conducted using small-angle X-ray scattering (SAXS) and circular dichroism (CD). The SAXS profile of LF (Figure 3A), represented by the scattering intensity *I*(*q*) versus scattering vector modulus (*q*), exhibits a characteristic pattern indicative of globular-shaped structures in solution. The pattern shows a plateau at *q* < ∼0.2 nm^–1^ and a Porod decay at *q* > ∼0.5 nm^–1^. The starting plateau and the subsequent decay of *I*(*q*) at *q* < ∼0.5 nm^–1^ were approximated using the Guinier equation to determine the radius of gyration (*R*_g_) of LF. The corresponding Guinier plot is shown in the inset of Figure 3A. The plot estimates *R*_g_ of LF from the slope of ln­(*I*) versus *q* ^2^, yielding an *R*_g_ value of 3.64 ± 0.13 nm. The linearity observed over a relatively large q range in the Guinier plot further supports the absence of large aggregates, consistent with the DLS results from the sonicated samples.

**Figure 3:**
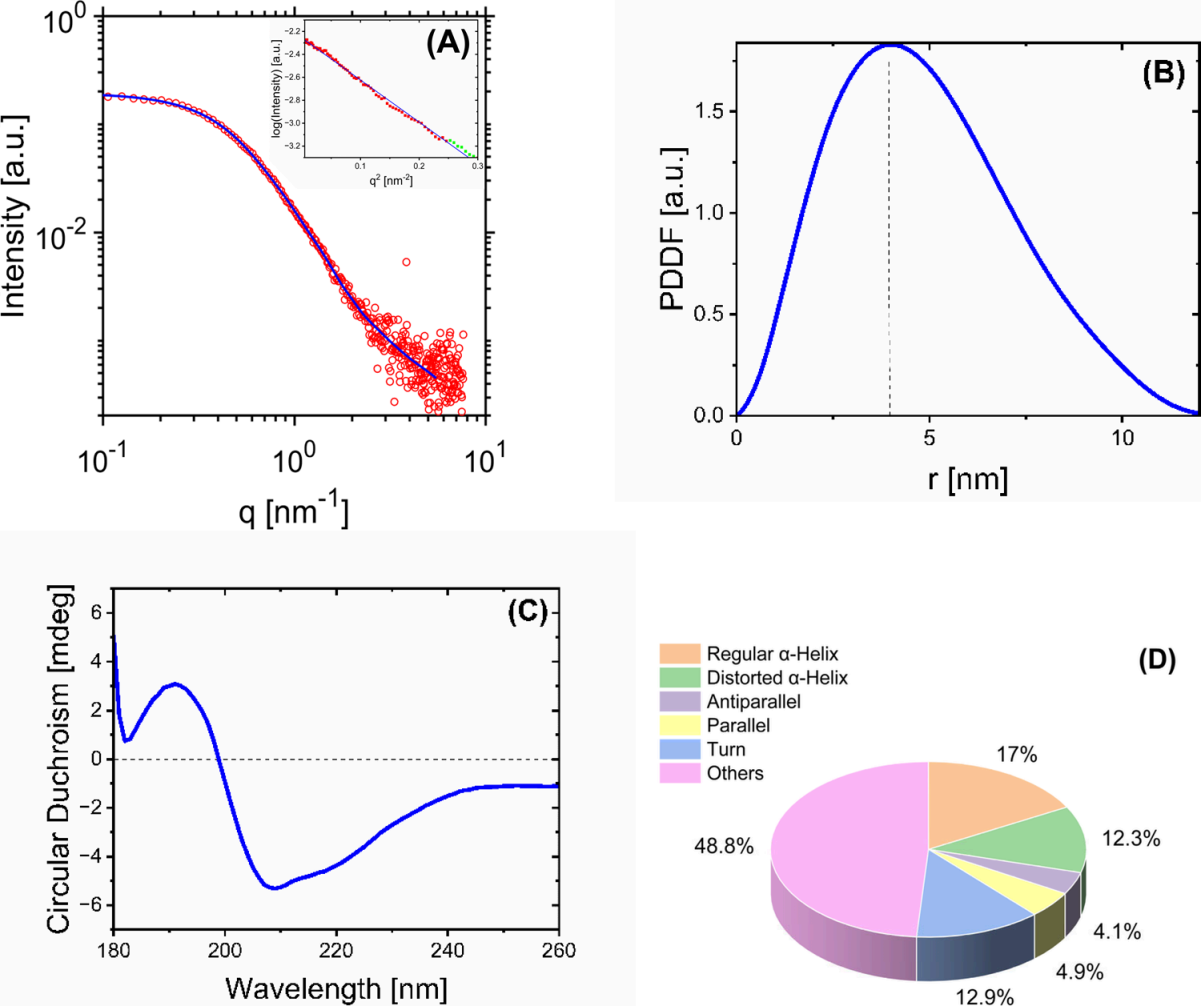
(A) SAXS profile of 1 wt % aqueous LF solution at pH 4. The blue line indicates the theoretical curve calculated by IFT, and the red circles present the experimental data. The inset shows the Guinier plot (ln­(*I*) versus *q* ^2^), where the slope of its linear interpolation resulted in the calculation of *R*_g_ = 3.64 ± 0.13 nm. (B) PDDF extracted from the IFT analysis. The vertical dashed line at 3.69 ± 13 nm marks the *R*_g_ obtained from the PDDF. (C) CD spectrum of 1 wt % LF solution. (D) Secondary structure composition of LF including α-helices, β-sheets, and turns. The category labeled “Others” comprises disordered regions and irregular structures of the protein, including the 3_10_-helix, π-helix, β-bridge, bend, and loop/irregular, as well as invisible regions of the LF structure.^99^

The exact size and shape of LF were determined using the more advanced pair-distance distribution function (PDDF) analysis of the SAXS data. The PDDF was calculated by the model-free indirect Fourier transformation of scattering intensities as a function of *q*, first introduced by Glatter.^87^ PDDF (Figure 3B) represents a histogram of distances within the protein particles, offering an exact calculation of *R*_g_ as well as the overall shape and maximum dimension of the LF particles (*D*_max_) in the protein solution. The maximum observable dimension by SAXS is set by the instrument resolution (*D*_res_), determined by the minimum reliable *q* measured experimentally (*q*_min_):^93,94^

3 $$D_{\mathrm{res}}=\frac{\pi }{q_{\min}}$$

When large particles are present in a sample (larger than the instrument resolution), the particles’ dimension goes beyond *D*_res_, leading to an underestimation of particle sizes by SAXS. In our LF analysis, shown in Figure 3B, the PDDF exhibits a bell-shaped function with a slight skewness toward larger sizes (*r*). This shape for the distribution function and its skewness suggests a deviation from a perfect sphere and the existence of more oblate-shaped protein particles. The skewness in the PDDF could also be associated with the presence of low concentrations of larger particles from partially agglomerated LF molecules that still exist, despite the ultrasonic pretreatment and filtration.^95^ The PDDF analysis also provides a more precise estimate of *R*_g_ for monomeric LF, at 3.69 ± 0.13 nm, which compares favorably with the Guinier-extracted value and also agrees with the established diameter of monomeric LF reported in previous studies.^31,91,96^

A complementary assessment of protein shape in solution can be considered by the ratio of the radius of gyration to the radius of hydrodynamic, *R*_g_/*R*_h_ (*R*_h_ represents the effective radius of the molecule during its diffusion, while *R*_g_ relates to the weighted distribution of mass averaged from all distances from the center of mass^97^). Taking the *R*_h_ from the first peak of DLS size distribution (3.8 ± 0.1 nm) and the *R*_g_ from SAXS (3.69 ± 0.13 nm), we obtain the *R*_g_/*R*_h_ ratio as 0.98. This ratio deviates slightly from that of the perfect spheres (*R*_g_/*R*_h_ = $\sqrt{3/5}\gg 0.77$ )^98^ yet demonstrates globular protein with slightly elongated shapes.

CD spectroscopy (Figure 3C) provided insights into LF secondary structures on a subnanometer scale, where the existence of α-helices and β-sheets was determined for the protein sample after ultrasonication. The CD pattern for LF showed a distinct positive absorption at 191 nm and a prominent negative absorption at ∼209 nm, both of which are characteristic of α-helical structures.^100^ The spectrum is very similar to that obtained elsewhere without further treatment of the protein solution, e.g., ultrasonication,^101^ suggesting that sonication itself did not significantly alter the secondary structures of the protein. The secondary structure composition of LF was quantified using the BESTSEL^29,99^ method within the wavelength range of 180–250 nm (Figure 3D). The results of the quantitative analysis for LF are in good agreement with those reported in a previous study by Gajda-Morszewski et al.^102^ In summary, all structural analyses indicate that our ultrasonic treatment of aqueous LF solutions at pH 4.0 resulted in a predominantly monomeric and stable protein solution, without any evidence of protein denaturation or alterations in its secondary structures.

### Pickering Mesosomes Stabilized by LF

The pretreated LF at pH 4 was used to formulate emulsions, aiming to utilize the protein as a solid-like particle stabilizer at the LLC–water interface and to enable the formation of highly stable Pickering emulsions. While the authors do not rule out the possibility that proteins may undergo partial unfolding at the emulsions interface, it is unlikely that LF in our formulation completely fails to function as a Pickering stabilizer. First, using X-ray reflectometry, Yano reported that LF only partially unfolds upon adsorption at the air–water interface.^103^ They showed that hydrophilic peptide chains extend into the aqueous phase, leaving the hydrophobic domains intact but exposed and oriented toward the air. Second, the internal phase employed in our LLC emulsion formulation is not a conventional hydrophobic oil, but rather the amphiphilic mesophases of monoglyceride-based self-assemblies in water. For instance, the *Pn*3*m* cubic phase is composed of amphiphilic GMO molecules and 25–40% (w/w) water.^104,105^ Compared to pure oil, this amphiphilic phase exhibits a lower interfacial tension with water, reducing the need for LF to unfold at the interface. Third, the LLCs exhibit high interfacial viscosity due to their self-assembled nanostructures, making the unfolding and extension of LF peptide chains less favorable compared to the air–water interface. Throughout the remainder of this paper, we will refer to the LF-stabilized Pickering emulsions containing various LLC mesophases as *lactoferrosomes*.

The stabilizing function of LF was confirmed by its adsorption at the LLC–water interface by confocal microscopy. Figure 4 shows CLSM micrographs of lactoferrosomes corresponding to the four mesophases studied (ME, *Fd*3*m*, H_2_, and *Pn*3*m*). The internal lipid phase was labeled with Nile Red, appearing yellow in the micrographs, while the proteins labeled with Fast Green appeared orange. All droplets were well dispersed and exhibited a spherical or nearly spherical shape. The faint uniform covering of orange spots suggests adsorption of LF at the interface. We note that, for CLSM experiments, the emulsions were fabricated by intentionally applying a reduced homogenization time (30 s) compared to the homogenization time used in all other experiments (6 min). The reduced homogenization resulted in micrometer-sized mesosomes that are large enough to be observed via CLSM. Otherwise, with a longer homogenization time, the droplet size decreases to the sub-micrometer range (as demonstrated in DLS experiments) and cannot be visualized under CLSM microscopy.

**Figure 4:**
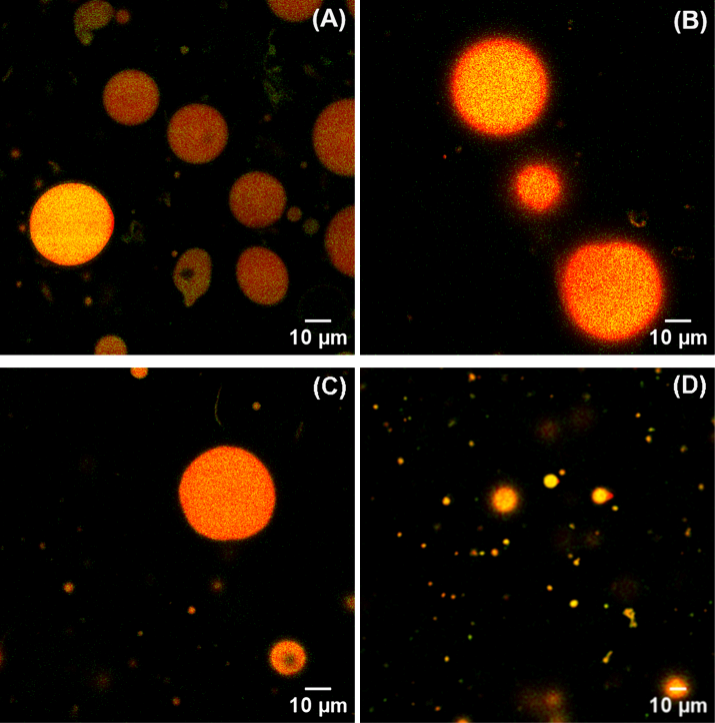
Representative CLSM micrographs of the lactoferrosomes of four different mesosomes: (A) ME, (B) *Fd*3*m*, (C) H_2_, and (D) *Pn*3*m*. Protein appears orange (labeled with Fast Green), and lipid appears yellow (stained with Nile Red); the scale bar = 10 μm.

Figure 5 shows the intensity-weighted size distribution of the lactoferrosomes with the inverse *Pn*3*m* cubic internal phase. Other mesophases of lactoferrosomes exhibited similar size distributions (see Figure S2); however, slight variations in particle diameter, resulting from different internal mesophases, have been observed (see Table 1). The diagram represents an average particle diameter of 187 ± 2 nm and a sharp peak, indicating a monodisperse lactoferrosome system. The visual appearance of the sample was slightly bluish, a characteristic typical of this particle size range when illuminated by natural light.

**Figure 5:**
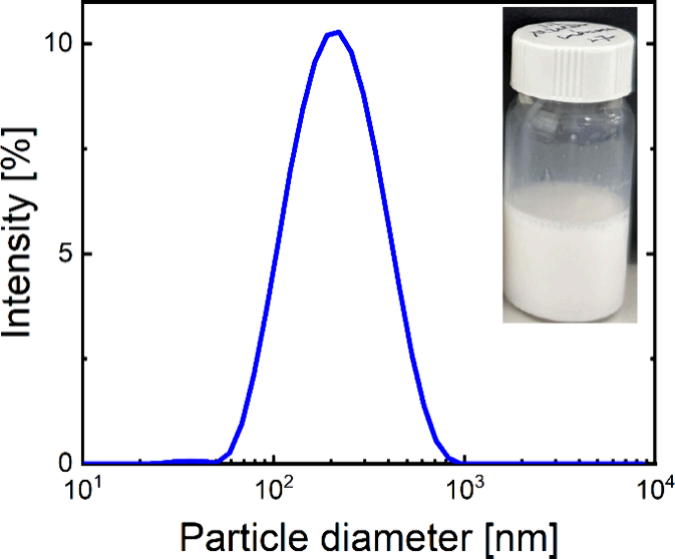
Representative intensity-weighted size distribution of lactoferrosomes obtained by homogenization of mesophases in the presence of LF by ultrasonication for 6 min. The inset shows a photograph of the dispersion with a milky appearance. The diagram illustrates the size distribution of cubosomes (lactoferrosomes within the internal *Pn*3*m* cubic phase) with an average particle diameter of 187 ± 2 nm.

### Internal Structure of Lactoferrosomes

The internal structures of lactoferrosomes with four distinct LLC phases were characterized by SAXS, as shown in Figure 6.

**Figure 6:**
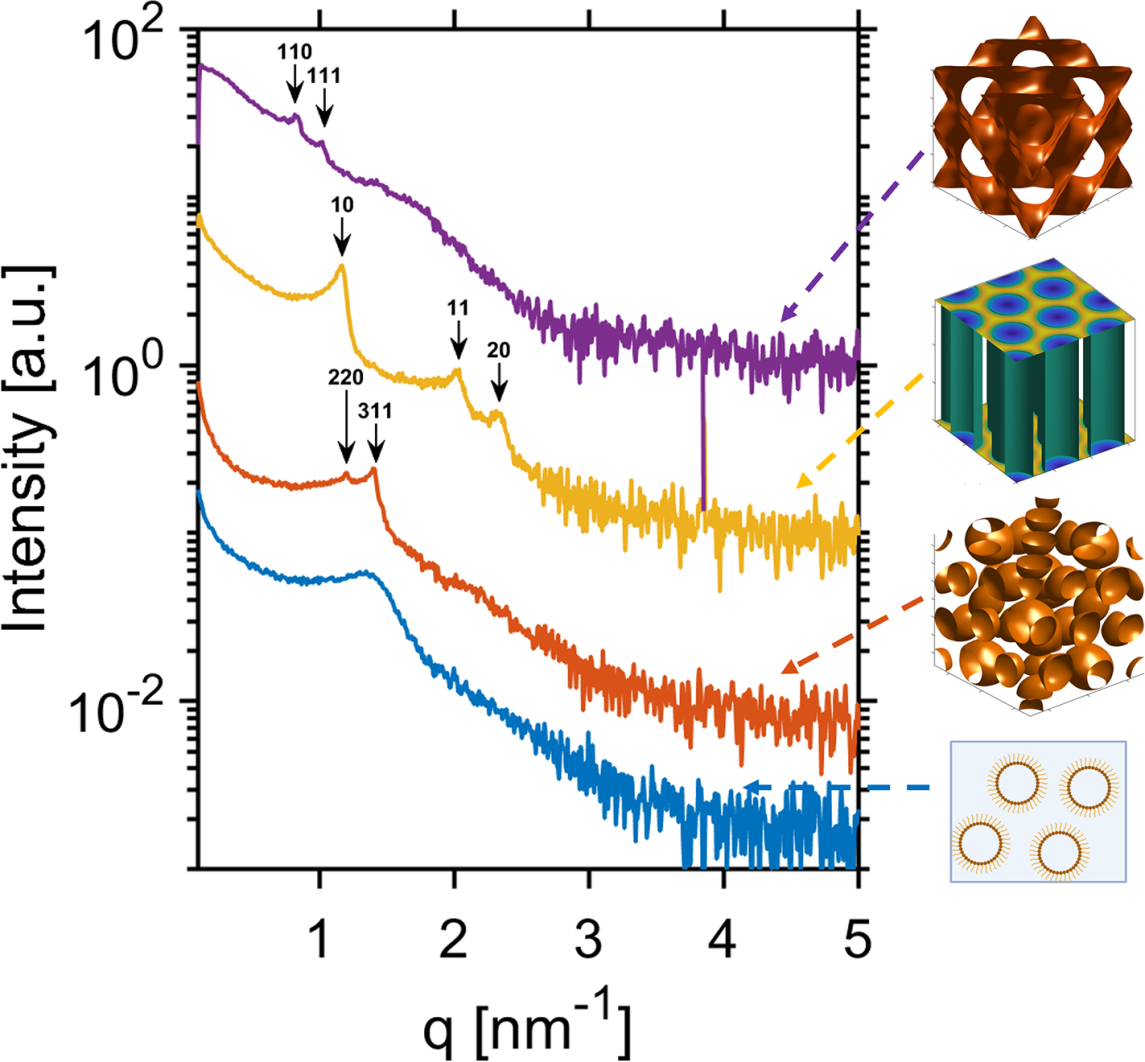
SAXS curves obtained from lactoferrosomes with four different internal phases prepared at various δ values: δ = 100 (*Pn*3*m*, purple); δ = 90 (H_2_, yellow); δ = 60 (*Fd*3*m*, orange), and δ = 54 (ME, blue). The images on the right-hand side of the scattering profiles depict the three-dimensional representation of the self-assembled LLC structures at the interior of lactoferrosomes. The internal phases shown for *Pn*3*m*, H_2_ and *Fd*3*m* are the electron density maps reconstructed from SAXS data, whereas the structure shown for ME is a schematic cartoon.

LLCs are characterized by their interfacial curvature, which is determined by the molecular geometry of their constituent amphiphilic molecules. For instance, GMO alone exhibits a double-wedge shape molecular geometry at the oil–water interface. Hence, GMO is suitable for forming saddle-like interfaces with slightly negative curvature and consequently self-assembles into a cubic mesophase. The gradual addition of OA into GMO results in the expansion of the hydrophobic side of the interfacial layer as OA positions near the hydrocarbon chains (the hydrophobic part) of GMO.^8,85^ Thereby, the interface further curves to more negative values, templating a change from a saddle-like to a cylindrical interface. Such curvature drives a phase transition in the self-assembling system from a cubic to a hexagonal mesophase.^6,106^ Further addition of OA enhances this curvature effect, leading to the formation of the *Fd*3*m* phase and then the microemulsion phase. In this study, we successfully fabricated 10 wt % lactoferrosomes with four distinct internal phases by fine-tuning the GMO-OA mixing ratio. Each of these internal phases represents a liquid crystalline structure with lattice parameters (LP) summarized in Table 1. The LPs obtained from our SAXS measurements are in good agreement with findings reported in previous studies on similar mesophases.^1,9,79^

The SAXS profiles confirm that LF can successfully stabilize all four mesophases to form lactoferrosomes, without inducing any phase transition in their internal structure. This apparent versatility of LF could therefore be particularly advantageous for potential applications in drug delivery,^107,108^ where different internal nanostructures may be necessary for optimal encapsulation and subsequent release of bioactive compounds.

### Stability of Lactoferrosomes

DLS measurements were conducted to assess the stability of lactoferrosomes against coalescence and aggregation over one month after preparation and storage at room temperature. The results, as depicted in Figure 7 for the ME, *Fd*3*m*, H_2_, and *Pn*3*m* mesosomes, demonstrate that the droplet size remains constant over the observation period. The fully stabilized ME and *Fd*3*m* mesosomes exhibited droplet diameters of 221 ± 2 and 239 ± 3 nm, respectively. In comparison, the H_2_ and *Pn*3*m* lactoferrosomes showed slightly smaller diameters of 201 ± 3 and 187 ± 2, respectively (see Table 1). All four mesosome formulations were initially designed to stabilize 10 wt % of the LLC phases. However, after homogenization, analysis revealed that a small fraction of H_2_ and *Pn*3*m* phases remained nonstabilized, as evidenced by the presence of residual lumps in their dispersions. This observation suggests that the incorporation of GMO/OA into the interior of the mesosomes is incomplete. By accounting for the mass of these residual lumps, the corrected stabilized LLC content was determined to be 9.5 wt % for H_2_ and 8.7 wt % for *Pn*3*m* lactoferrosomes. This reduced LLC content may also account for the notable decrease in droplet size observed in H_2_ and *Pn*3*m* formulations.

**Figure 7:**
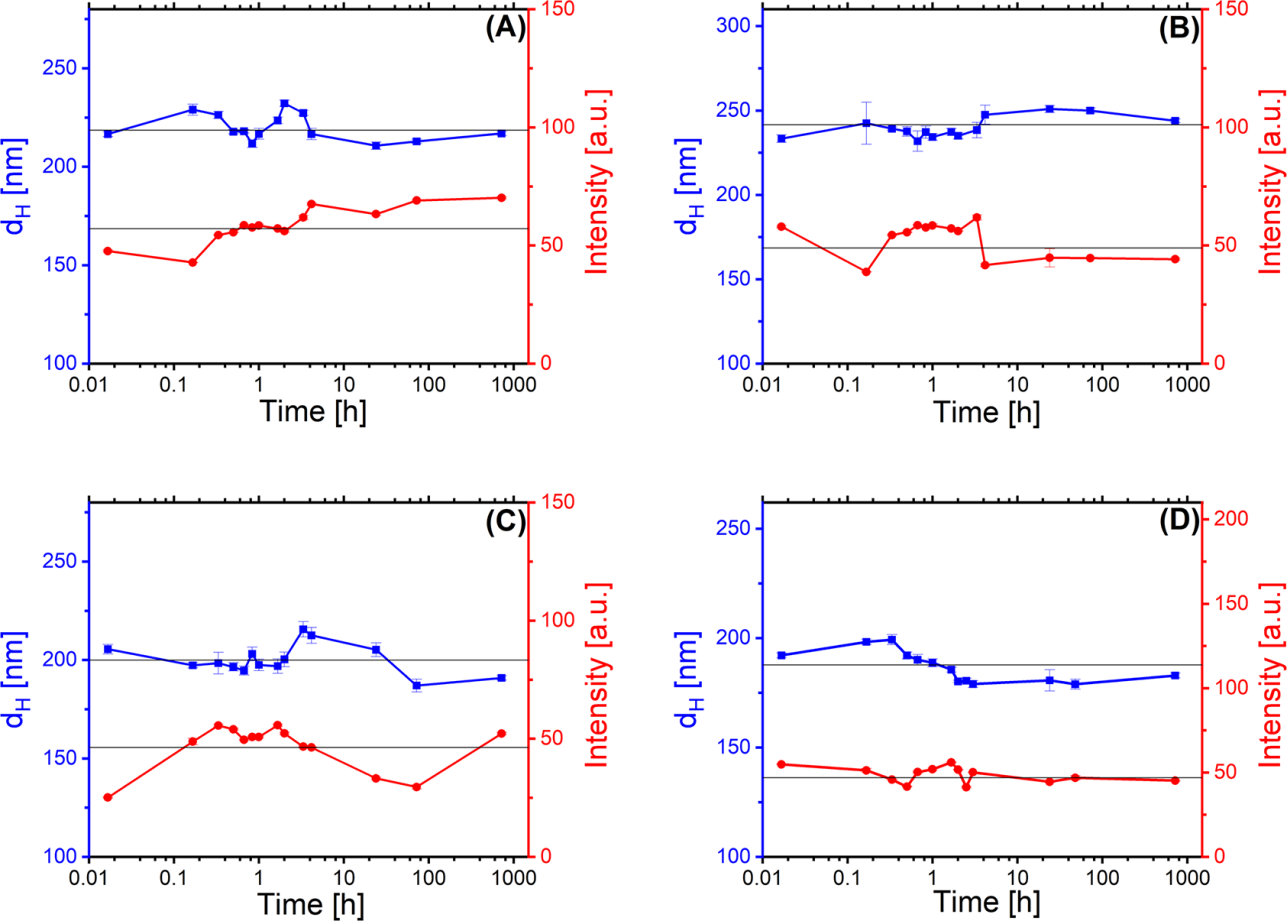
Hydrodynamic diameter (*d*_H_) of ME (A), *Fd*3*m* (B), H_2_ (C), and *Pn*3*m* (D) lactoferrosomes, determined by DLS over 1 month after preparation and storage in water at 25 °C. The *d*_H_ data are presented by blue squares (■), and the scattering intensities are shown by red circles (●).

To rule out any systematic errors in size evaluation due to creaming over the long storage period, we monitored the average scattering intensity of the samples alongside their size measurements. A consistent scattering intensity measured for all emulsion droplets throughout the observation period indicated that the concentration of emulsions also remained unchanged; i.e., there was no considerable creaming of the droplets. The PDI remained consistent at around 0.2 (see Figure S3), and the monomodal intensity-weighted size distribution observed throughout the 1 month (see Figure S2) closely matched that of the freshly prepared samples. In summary, our long-term stability study using DLS indicates that the lactoferrosome systems remained highly stable with no significant signs of aggregation, coalescence, or Ostwald ripening observed over one month of storage at room temperature.

## Conclusions

In this study, we demonstrated the engineering of lactoferrin-stabilized emulsions (lactoferrosomes) containing lyotropic liquid crystalline (LLC) phases, characterized by droplet diameters of approximately 200 nm and a positive surface charge. The LF structure was characterized before emulsification, and discussions on possible LF conformational changes upon adsorption at the LLC–water interface were provided. We concluded that at most, LF is only partially unfolded at the LLC–water interface of our formulation; hence, the engineered emulsions were referred to as Pickering mesosomes.

The lyotropic phases that form emulsion droplets were comprised of four different GMO-based self-assembled mesophases, namely, microemulsions (ME), *Fd*3*m* cubic phase, inverse hexagonal phase (H_2_), and *Pn*3*m* bicontinuous cubic phase. Prior to emulsification, ultrasonication was applied as a pretreatment to disintegrate LF agglomerates, resulting in a predominantly monomeric LF solution suitable for use as a Pickering stabilizer. The results from SAXS, time-resolved DLS, and electrokinetic experiments demonstrate that our proposed emulsification approach preserves the original architecture of the mesophases in the droplets and provides them with good temporal stability against coalescence, creaming, and aggregation at least over one month. The stabilization of LLCs by LF suggests advantages over Pluronic block copolymer F127, which is commonly used in the manufacture of LLC-based emulsions. First, our formulated lactoferrosomes carry a positive surface charge, arising from LF molecules adsorbed at the interface, which confers high stability under acidic conditions. Although not directly investigated in this study, it is anticipated that the stability of lactoferrosomes may be compromised at elevated pH levels. This suggests that the formulation is particularly suitable for controlled drug release under neutral pH conditions. Second, in the case of stabilized GMO (δ = 100), the internal phase of the lactoferrosomes retains its *Pn*3*m* symmetry, consistent with its bulk behavior. This contrasts with stabilization by F127, which induces a phase transition to *Im*3*m* symmetry.

Positively charged LLC mesosomes with pH-responsive surface properties and long-term stability offer several advantages for the design of advanced colloidal systems. These include lactoferrosomes’ ability to encapsulate both hydrophilic and lipophilic molecules within their internal LLC phases, thus enabling pH-responsive release and facilitating targeted drug delivery through interactions with negatively charged macromolecules in the gastrointestinal tract, such as mucins in the mucosa. Further research is required to understand their encapsulation efficiency, in vitro digestibility, and interactions with other molecules from the food matrix and digestive system.

## Supplementary Material



## Notes

Details of lattice parameter calculation and related images (Figure S1); intensity-weighted size distribution and polydispersity index for lactoferrosomes (Figures S2 and S3); SAXS patterns for bulk phases (Figures S4); macroscopic images of lactoferrosomes (Figure S5) (PDF)
