# Supplementary material for: Internally Self-Assembled Pickering Mesosomes Stabilized by Positively Charged Lactoferrin
Source: Langmuir. 2025 Nov 3;41(45):30090–101. doi: 10.1021/acs.langmuir.5c02484 (PMC12632182; doi:10.1021/acs.langmuir.5c02484)
Supplement: Supplementary file 1 [file la5c02484_si_001.pdf]

Supporting information

Internally Self-assembled Pickering Mesosomes Stabilized by Positively Charged Lactoferrin

Yi Li [a], Brent S. Murray [a], Célia Ferreira [a], Francisco M. Goycoolea [a,b] and Amin Sadeghpour [a]*

[a] Food Colloids and Bioprocessing Research Group, School of Food Science and Nutrition, University of Leeds, Leeds, West Yorkshire, LS2 9JT, United Kingdom

[b] Department of Cell Biology and Histology, Faculty of Biology, University of Murcia, Campus de Espinardo, Murcia, 30100, Spain

This PDF file includes:

Pages S1 to S7

Figures S1 to S5

Table of contents:

**Figure S1**	The linear fittings of observed q versus theoretical q_{peak} values
**Figure S2**	Lactoferrosome emulsions droplet size distributions immediately after preparation compared to one month after preparation
**Figure S3**	The change in polydispersity index (PDI) of emulsion droplets over one month storage
**Figure S4**	SAXS profiles of lyotropic liquid crystalline phases in bulk
**Figure S5**	Macroscopic pictures showing the visual appearance of lactoferrosome emulsions

*Corresponding Author:
E-mail address: a.sadeghpour@leeds.ac.uk (Amin Sadeghpour)

Lattice parameter

Small-angle X-ray scattering (SAXS) patterns exhibited distinct Bragg peaks, indicating the presence of three internal phases: Pn3m, H_2, and Fd3m. In addition, ME was observed in a pattern with a q_{max} value feature. To confirm the internal nanostructures of the ordered phases, the Miller indices (h, k, l) were assigned to each diffraction peak. For the H_2 phase, reflections corresponding to the [10], [11], and [20] were identified, with relative q ratios of 1, $\sqrt{3}$ and $\sqrt{4}$, respectively. These ratios are consistent with the expected diffraction pattern of a H_2, confirming its structural identity.

In self-assembled nanostructures, the lattice parameter represents the periodic distance between adjacent unit cells. For the H2 phase, the observed q values of 1.384 nm^{-1}, 2.407 nm^{-1}, and 2.784 nm^{-1} were plotted against the square roots of the corresponding Miller indices. The slope of the resulting linear fit was calculated as 1.391 nm^{-1} using Excel. The lattice parameter was then determined to be 4.51 nm based on the equation $2\pi/slope$. The standard error of the slope, calculated using Excel's LINEST function, was found to be 0.002 nm.

For the Pn3m phase, Bragg peaks were observed at q-values of 0.905 nm^{-1} and 1.115 nm^{-1}, corresponding to relative q-spacing ratios of $\sqrt{2}$ and $\sqrt{3}$. The slope of the linear fit was 0.643 nm, yielding a lattice parameter of 9.77 nm, with a standard error of 0.0006 nm. Similarly, the Fd3m phase exhibited peaks at 1.186 nm^{-1} and 1.391 nm^{-1}, associated with relative q-spacing ratios of $\sqrt{8}$ and $\sqrt{11}$. Using the same approach, the calculated slope resulted in a lattice parameter of 14.9 nm, with a standard error of 1.5×10^{-5} nm.

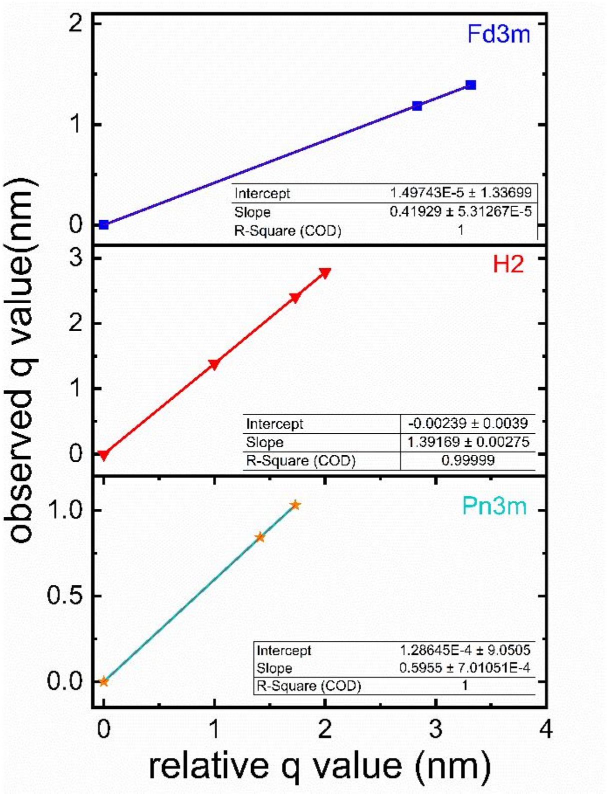

Figure S1. Linear fitting of observed q-values versus relative q-values for three internal mesophases identified by SAXS: Fd3m (top), H2 (middle), and Pn3m (bottom). Each plot includes the slope, intercept, and coefficient of determination (R^2), data were analyzed by OriginPro 2024.

Intensity-weighted size distribution of Lactoferrosomes

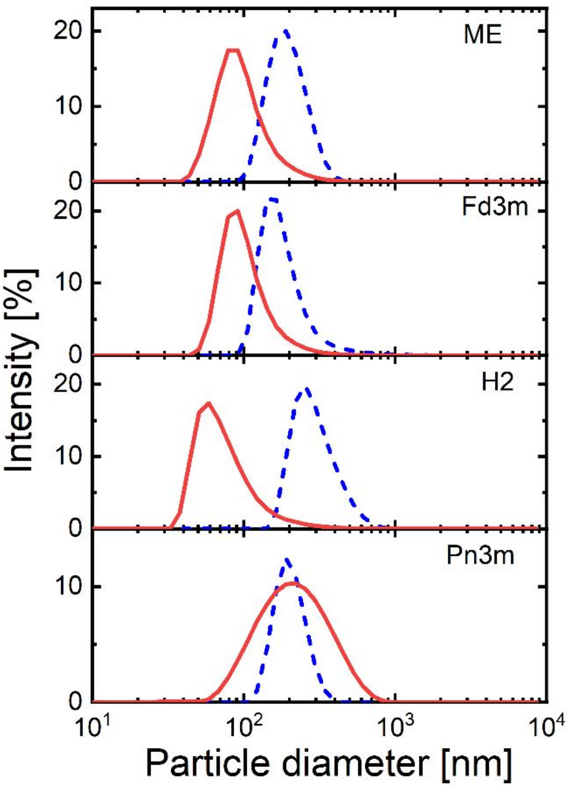

Figure S2. The intensity-weighted size distribution at the initial time point (red solid line) and after one month of storage (blue dashed line) for ME, Fd3m, H2, and Pn3m from top to bottom.

Polydisperse Index (PDI) of Lactoferrosomes

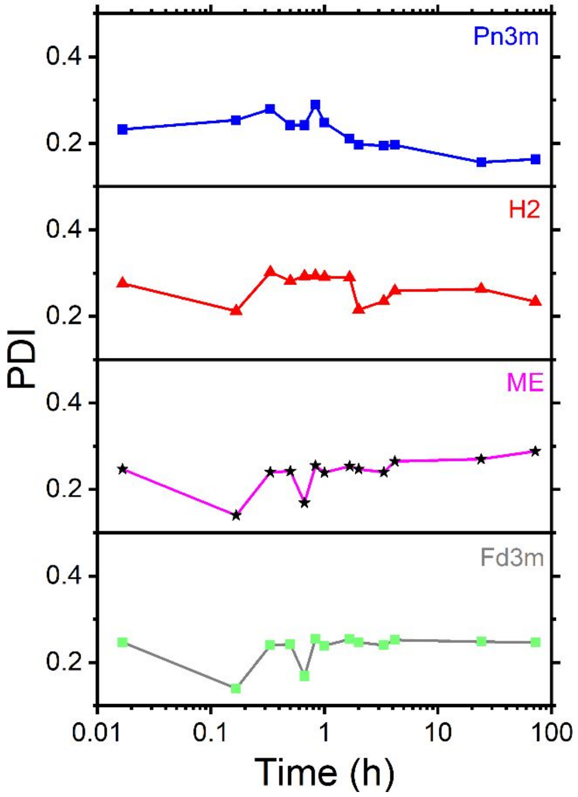

Figure S3. PDI as a function of time for: Pn3m (blue), H2 (red), ME (magenta), and Fd3m (grey), from top to bottom, the measurements were conducted over a logarithmic time scale.

Bulk phases

The SAXS profiles presented correspond to the bulk phases of the respective samples. Each sample exhibits scattering features characteristic of its assigned bulk phase. The microemulsion (ME) sample, which does not form a bulk phase, has therefore been excluded from here.

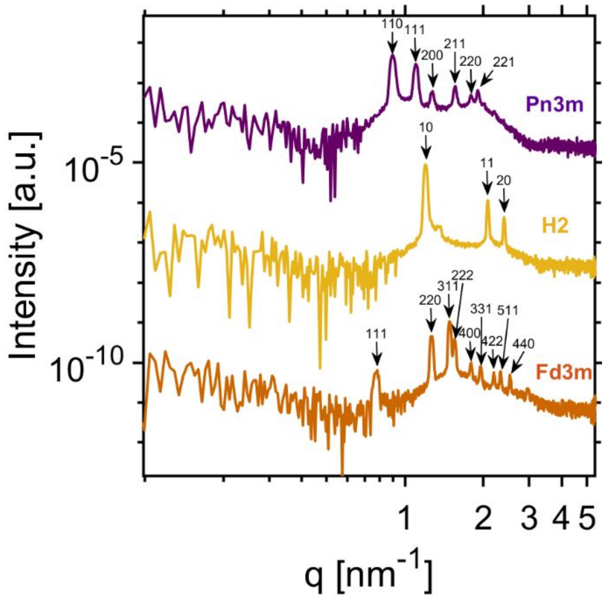

Figure S4. SAXS curves for bulk phase, Pn3m(purple); H2(yellow), and Fd3m(orange) with labelled Miller indices, from top to bottom.

Macroscopic Images of Lactoferrosomes

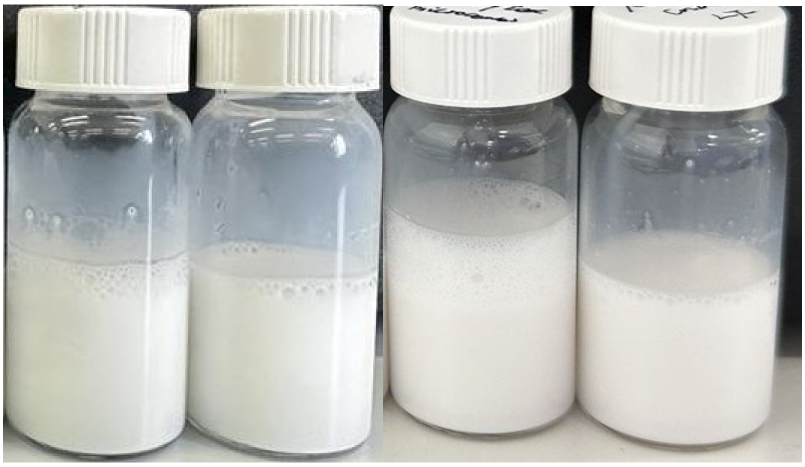

Figure S5. The macroscopic images of the Lactoferrosome were captured after modification. These images provide visual confirmation of the physical stability of the various phases. From left to right are Fd3m, ME, H2, and Pn3m, respectively.
